# Positive Pattern Recognition System Using Alanine Aminotransferase, Type IV Collagen 7s, and E Value (Liver Stiffness) for the Diagnosis of Nonalcoholic Steatohepatitis Based on Natural History

**DOI:** 10.31662/jmaj.2021-0199

**Published:** 2022-03-31

**Authors:** Masayuki Tsujisaki, Shigeru Sasaki, Noriyuki Akutsu, Takenori Takamura, Tetsuyuki Igarashi, Mitsuru Yoshimoto, Hideto Itoh, Yoshio Kurihara, Hiroshi Nakase

**Affiliations:** 1 Department of Gastroenterology, Tenshi Hospital, Sapporo, Japan; 2 Department of Gastroenterology and Hepatology, Sapporo Medical University, Sapporo, Japan; 3 Department of Hematology, Tenshi Hospital, Sapporo, Japan; 4 Kurihara Naika Clinic, Sapporo, Japan

**Keywords:** nonalcoholic fatty liver disease, natural history, positive pattern recognition system

## Abstract

**Introduction:**

The use of a simple diagnostic system for nonalcoholic fatty liver disease (NAFLD) instead of a biopsy is expected. We investigated a positive pattern recognition system for the evaluation of nonalcoholic fatty liver (NAFL) and the stages of nonalcoholic steatohepatitis (NASH).

**Methods:**

A total of 68 Japanese patients with biopsy-confirmed NAFLD were enrolled. Serological biomarkers and medical imaging markers were investigated to determine candidate markers. The markers were statistically evaluated, and the patients were distributed to pattern combinations.

**Results:**

We selected three markers based on natural history and set the critical values: alanine aminotransferase/ALT (persistent ≧ 44 IU/L) as a marker for hepatitis, type IV collagen 7S (≧5.1 ng/mL) for fibrosis, and E value (≧5.5 kPa) for stiffness. After evaluation of statistical accuracies, every patient was classified into their combination patterns. Comparing the relationships between histological classifications and positive patterns, the patients with NAFL were mainly distributed in pattern (ALT, type IV collagen, E value: −, −, −), those with NASH stage 0-1 in (+, −, +), those with NASH stage 2-3 in (+, +, +), and those with NASH stage 4 in (−, +, +).

**Conclusions:**

The positive patters changed with the NAFL and NASH conditions. Our results indicated a correlation between the positive patterns using three markers and the histological results. The positive pattern recognition system based on natural history is useful for the differential diagnosis of NAFLD and for the evaluation of the severity of fibrosis in patients with NASH.

## Introduction

Due to the increase in the prevalence of diabetes, metabolic syndrome, and obesity over the past couple of decades, 20%-30% of the adult population is estimated to have suffered from nonalcoholic fatty liver disease (NAFLD) in both developed and developing countries ^(1)^. According to a nationwide survey, the major complications of diabetes did not only include vascular diseases but also liver diseases, especially hepatocellular carcinoma (HCC) and liver cirrhosis (LC). Thus, NAFLD has become one of the most common diseases and severe problems worldwide ^(2), (3)^. NAFLD includes a wide spectrum of liver diseases. The histological forms range from nonalcoholic fatty liver (NAFL), which is generally nonprogressive, to nonalcoholic steatohepatitis (NASH), which can progress to chronic hepatitis, LC, and sometimes HCC ^(4), (5)^.

Liver biopsy remains a reliable method for the diagnosis of NASH, and it is recommended as a gold standard for staging and grading ^(6), (7)^. However, this procedure is invasive, poses risks, and complications, causes sampling error, and is practically difficult to perform for every patient due to the large number of NAFLD patients. Therefore, a simple, noninvasive system to distinguish NASH from NAFLD and determine the stage of NASH needs to be developed. Many scoring systems and a new trial to identify multi-biomolecules for immunological responses and for diagnostic tools by data mining have been reported ^(8)^. This study aimed to investigate the clinical markers and to develop a simple system for the diagnosis of liver fibrosis and for the evaluation of its severity in patients with NASH. To explore and select the potential clinical markers, we focused on state of inflammation, fibrosis, and diagnostic imaging along with the natural history of NASH. Furthermore, we investigated the possibility of pattern recognition system for the diagnosis of NASH.

## Materials and Methods

### Study population and data collection

Of the 269 patients who had liver dysfunction or had been diagnosed with fatty liver, chronic hepatitis, or LC at Tenshi Hospital, Sapporo, Japan, between January 1, 2017, and July 31, 2020, 68 who had liver biopsy-confirmed NAFLD were included in this study. The exclusion criteria were the presence of chronic hepatitis B or C, autoimmune hepatitis, primary biliary cholangitis, primary sclerosing cholangitis, and Wilson’s disease and consumption of more than 30 and 20 g of alcohol per day in men and women, respectively.

For the body measurements, body mass index, visceral fat, and liver/spleen (L/S) ratio measured by CT imaging were used. Aspartate aminotransferase, alanine aminotransferase (ALT), fasting immunoreactive insulin (F-IRI), ferritin, type IV collagen 7S, hyaluronic acid, and platelets were selected as biochemical data.

The patients were assigned a diagnosis of lifestyle-related diseases. Dyslipidemia, hypertension and diabetes mellitus (DM) were diagnosed according to each diagnostic criterion.

Liver stiffness ^(9)^ was measured using FibroScan (Echosens, Paris, France). The vibration induces an elastic shear wave, and the propagation and velocity of the wave are measured *via* simultaneous ultrasonography and expressed in kilopascals (kPa). The median value referred to as E (elasticity) was determined as the liver elastic modulus. The details were presented in previous report ^(9)^. The controlled attenuation parameter (CAP) specifically targets hepatic steatosis using a process based on FibroScan. The final CAP value ^(10)^ was expressed in dB/m.

### Histological assessment

**Figure 1.**
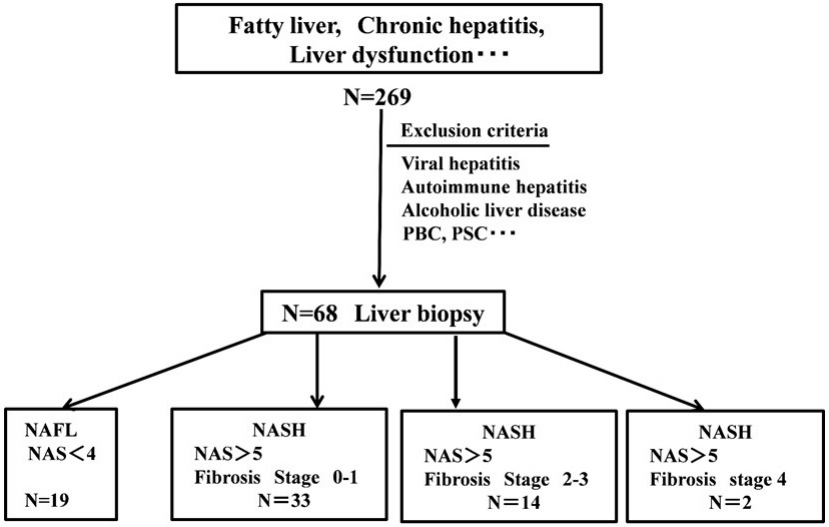
A schematic representation of the study design.

Consecutive patients with NAFLD (n = 68) undergoing liver biopsies were recruited. Histological scoring was performed by an expert pathologist without any information according to the NASH Clinical Research Network Scoring System ^(6)^. The disease severity was evaluated using the NAS (NAFLD activity score) as the unweighted sum of scores of steatosis, hepatocyte ballooning, lobular inflammation, and fibrosis. Furthermore, the Brunt staging was performed. After the histopathological evaluation, the liver histology, four groups were identified, and the patients were divided into four subgroups (Figure 1).

Subgroup 1 (NAFL): patients with simple steatosis or nonalcoholic fatty liver (NAFL = NAFLD but no NASH) (n = 19)

Subgroup 2 (NASH): patients with NASH without advanced fibrosis (fibrosis score 0-1) (n = 33)

Subgroup 3 (NASH with fibrosis): patients with NASH with advanced fibrosis (fibrosis score 2-3) but no cirrhosis (n = 14).

Subgroup 4 (NASH with LC): patients with NASH with cirrhosis (fibrosis score 4) (n = 2)

### Statistical analysis

The statistical differences between NAFL and NASH were determined using the *t*-test for quantitative data. Multivariate analysis was conducted using logistic regression to independently identify variables associated with the presence of NASH and those associated with the natural history of NASH. We calculated the sensitivity and specificity to evaluate the accuracy of the clinical scoring system in determining NASH and NAFL. Using these results, we constructed receiver operating characteristic (ROC) curves by plotting sensitivity against 1-specificity at each value. The diagnostic performance of the prediction models was evaluated by analysis of the ROC curves. The area under the ROC curve (AUROC) was used as the statistical measure of accuracy ^(11)^, with values close to 1.0 indicating high diagnostic accuracy. The sensitivity, specificity, positive predictive value (PPV), and negative predictive value (NPV) were calculated to evaluate the accuracy of the selected scores. *P* values were calculated *via* *t*-test, and the differences were considered to be statistically significant at *P* < 0.05.

### Approval by the institutional review board (IRB)

All procedures were in accordance with the ethical standards on human experimentation and with the Declaration of Helsinki 1964. This research was approved by the ethics committee of Tenshi Hospital (approval number: 147-2021; approval date: November 19, 2021). Although informed consent was not obtained from the participants, they were provided with the opportunity to deny participation by posting the opt-out document.

## Results

### Patient characteristics

Table 1 summarizes the clinical characteristics, body measurements, diagnosis of lifestyle-related disease, biochemical data, diagnostic imaging marker, E value, CAP measured using FibroScan, and liver/spleen (L/S) ratio measured *via* CT scan.

**Table 1.**
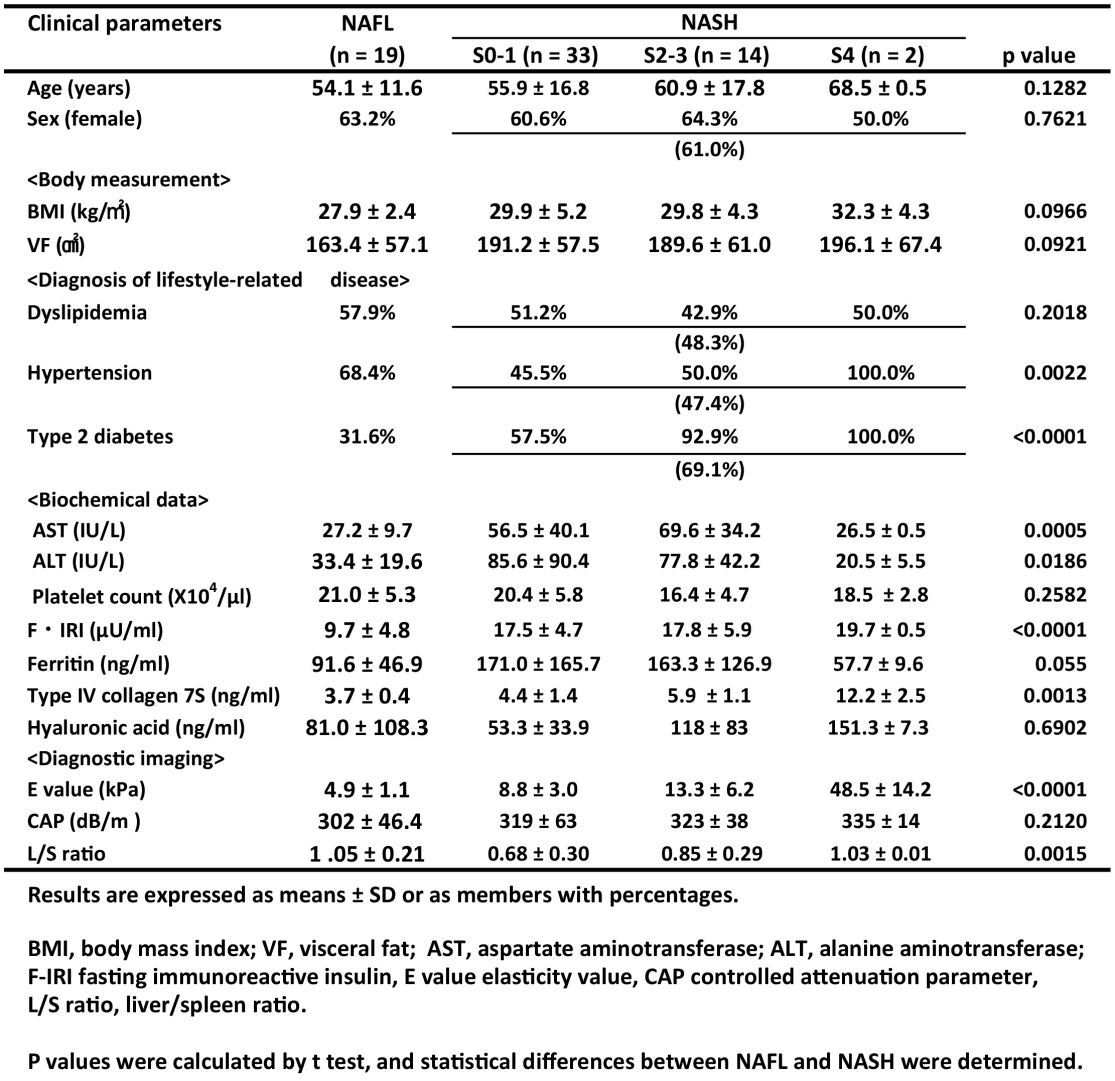
Clinical Characteristics of Patients with NAFL and NASH.

Statistical analysis revealed that type 2 DM, F-IRI, and E value were significant variables. Type 2 DM could not be a marker, and F-IRI would be useful for the diagnosis of NASH. However, in this study, we did not select either type 2 DM or F-IRI because these markers would not be parallel to liver histology.

### Selection of the predictors of NASH

We recommend that inflammation and fibrosis markers be selected as the predictors of NASH because histological imaging is certainly affected by them according to the natural history of NASH.

**Figure 2.**
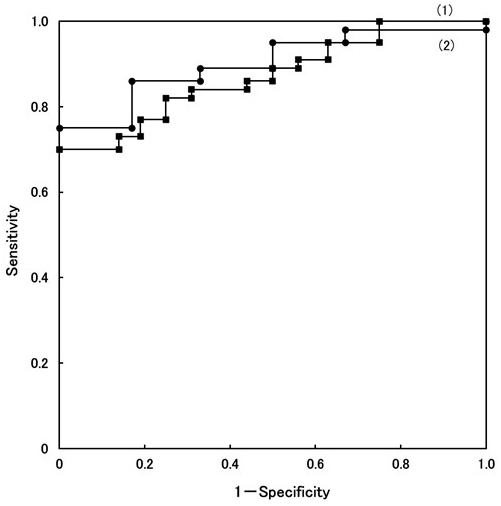
Receiver operating characteristic (ROC) analysis of ALT. The sensitivity and specificity of ALT were determined in the study to discriminate patients with NAFL from those with NASH s0-1/NASH s2-3 (1) as well as patients with NASH s0-1/NASH s2-3 from those with NASH s4/LC (2).

ALT is a marker of hepatitis level, but it is not sufficient for the differential diagnosis of all NASH from NAFL (*P* value 0.0186). However, we have encountered many NASH patients who exhibited persistently abnormal ALT level, NAFL patients showed low value, and also NASH LC patients showed low value. The ROC curve of ALT is presented in Figure 2. ROC curve (1) depicts the ability to discriminate between NAFL patients and NASH s0-1/NASH s2-3 patients. The cutoff value was set to 44 IU/L. The sensitivity and specificity were 81.8% and 75.0%, respectively. The *t*-test revealed a significant difference (*P* < 0.003) at a cutoff value of 44 IU/L.

On the other hand, the number of NASH s4 patients is small; thus, it is difficult to analyze the ROC curve to distinguish between NASH s0-1/NASH s2-3 and NASH s4 patients. Therefore, the ALT levels of four NASH/LC patients were added only for the analysis of the ROC curve. The character of four patients was as follows: patients have already diagnosed by liver biopsy before, Child Pugh classification B, C, and A, LC diagnosed by CT imaging, but it is difficult to undergo liver biopsy for the reasons of liver atrophy, ascites, and bleeding tendency.

ROC curve (2) depicts the ability to distinguish between NASH s0-1/NASH s2-3 and NASH s4/LC (n = 6), presented in Figure 2. At a cutoff value of 36 IU/L, the sensitivity and specificity were 86.3% and 83.4%, respectively (reference data). The *t*-test revealed a significant difference (*P* value < 0.041), presented in Table 2.

**Table 2.**
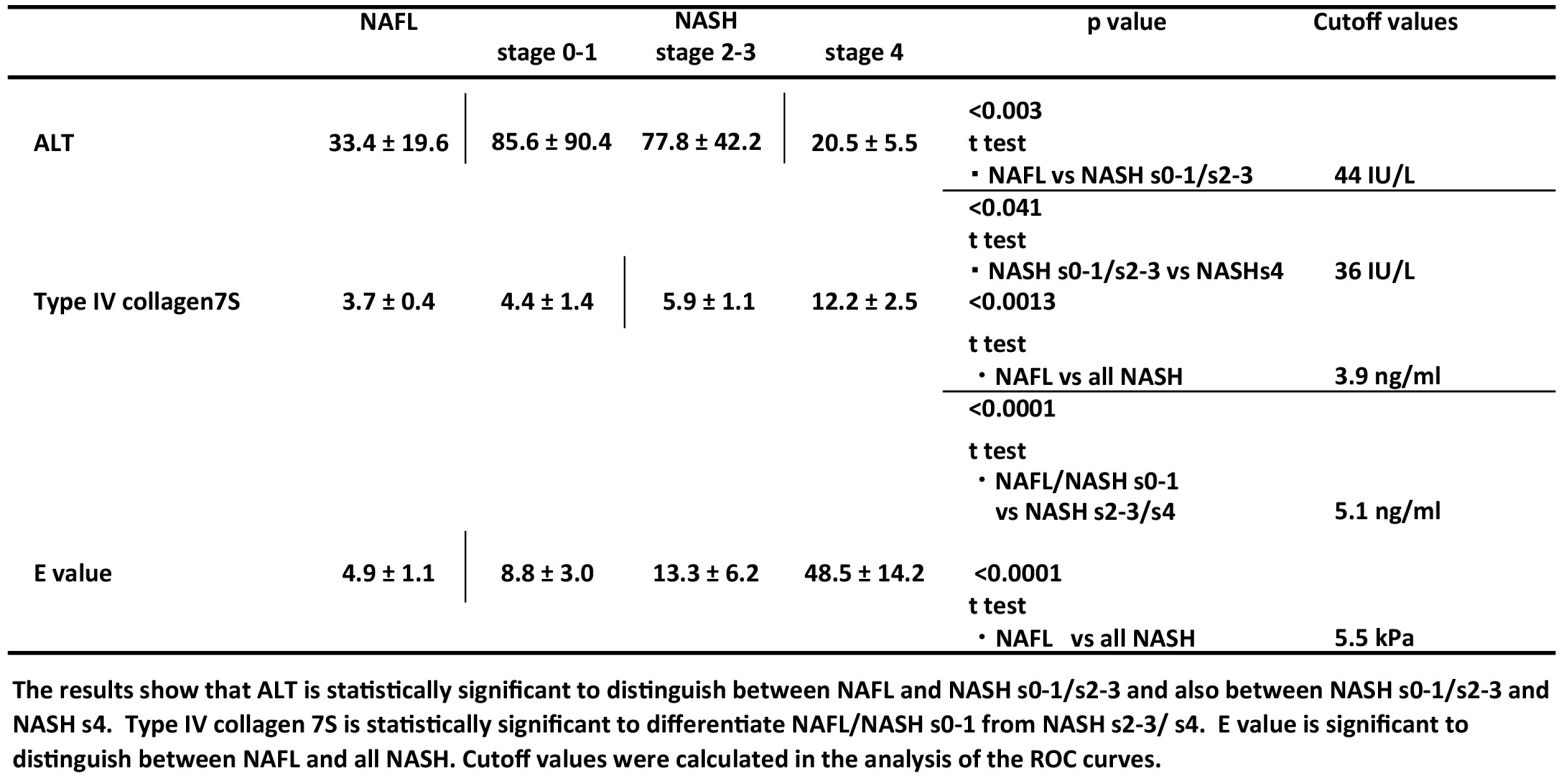
The Summary of Statistical Analysis.

Based on the result of the statistical evaluation, the cutoff value of ALT was set to 44 IU/L. The point is that the ALT level was almost normal, not only in NAFL patients but also in NASH s4/LC patients.

**Figure 3.**
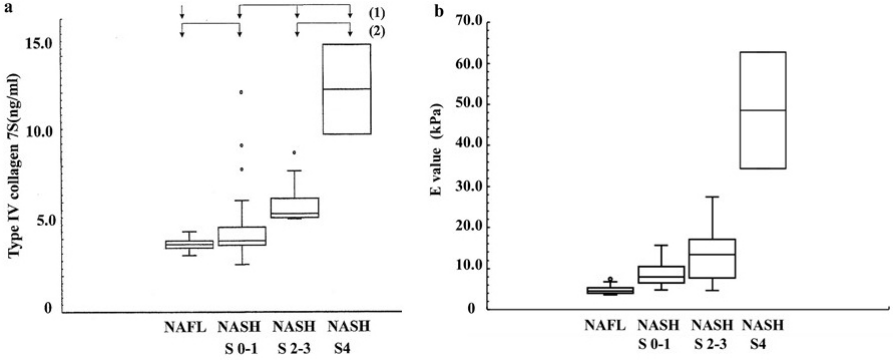
(a) Boxplots (median, upper, and lower quartiles, range, and outliers (circles)) of type IV collagen 7s. A stepwise increase in type IV collagen 7s was observed from NAFL to NASH s4. Note: (1): *P* < 0.0013 between NAFL and all NASH (2): *P* < 0.0001 between NAFL/NASH s0-1 and NASH s2-3/NASH s4. (b) Boxplots (median, upper, and lower quartiles, range, and outliers (circles)) of E value. A stepwise increase in E value was observed from NAFL to NASH s4. Note: *P* < 0.0001 between NAFL and all NASH.

Type IV collagen 7S is known to be a marker of liver fibrosis. However, the *t*-test did not obtain good results (*P* value = 0.0013) between NAFL and all NASH patients. It was demonstrated that the values of NASH s2-3 (5.9 ± 1.1 ng/mL) and NASH s4 (12.2 ± 2.5 ng/mL) were higher than those of NAFL (3.7 ± 0.4 ng/mL) and NASH s0-1 (4.4 ± 1.4 ng/mL) in Figure 3-a. The ROC curves were plotted (data not shown). The first ROC curve depicts the ability to discriminate between NAFL and all NASH patients. The AUROC was 0.823 at a cutoff value of 3.9 ng/mL, and it did not show a good *t*-test result.

According to the second ROC curve between NAFL/NASH s0-1 and NASH s2-3/NASH s4 (data not shown), the AUROC was 0.896 at a cutoff value of 5.1 ng/mL. The *t*-test revealed more significant difference (*P* value < 0.0001) (Table 2). As a result, the cutoff value of 5.1 ng/mL was used to discriminate between NAFL/NASH s0-1 and NASH s2-3/NASH s4/LC.

The E value (kPa) was selected as the third marker. It expresses the level of liver stiffness measured using FibroScan. Figure 3-b demonstrates that the distribution of the E values significantly increased from NAFL, NASH s0-1, NASH s2-3, to NASH s4 together with the fibrosis and/or chronic inflammation. The median liver stiffness values were as follows: NAFL, 4.9 ± 1.1; NASH s0-1, 8.8 ± 3.0; NASH s2-3, 13.3 ± 6.2; and NASH s4, 48.5 ± 14.2 kPa. The AUROC of the E value are plotted (data not shown). This curve depicts the ability to distinguish between NAFL and all NASH patients. The sensitivity, specificity, PPV, and NPV were 93.6%, 83.3%, 91.7%, and 82.4%, respectively, at a cutoff value of 5.5 kPa. The AUROC was 0.926. Furthermore, the *t*-test revealed a significant difference (*P* value < 0.0001) between NAFL and all NASH patients (Table 2).

From the results shown above, we selected three markers: (1) persistent abnormality of ALT level as an indicator of hepatitis; (2) type Ⅳ collagen 7S, liver fibrosis; and (3) E value, liver stiffness, including fibrosis and chronic inflammatory activity. These markers can complementarily detect the characteristics of NASH. The ACE system employs pattern recognition using three markers.

The pathological conditions of NASH change from fatty liver to chronic hepatitis, LC and hepatoma along a natural history. The positive patterns using three markers must change with the different state, because the medical conditions of NAFL and every stage of NASH reflect the difference in hepatitis, fibrosis, and stiffness.

### Positive pattern combinations using three markers (ACE system)

The total number of combinations is eight: ① (ALT, type IV collagen, E value: +, +, +), ② (+, +, −), ③ (+, −, +), ④ (+, −, −), ⑤ (−, +, +), ⑥ (−, +, −), ⑦ (−, −, +), and ⑧ (−, −, −).

A total of 14 NAFL patients showed negative ⑧, 19 NASH s0-1 patients showed positive ALT and E value ③, and 11 NASH s2-3 patients showed positive ①. Almost none of the patients showed positive type IV collagen 7S and E value negative ② and ⑥. Because the sensitivity of the E value was higher than that of type IV collagen 7S, both the ② and ⑥ groups could be deleted from Table 3.

**Table 3.**
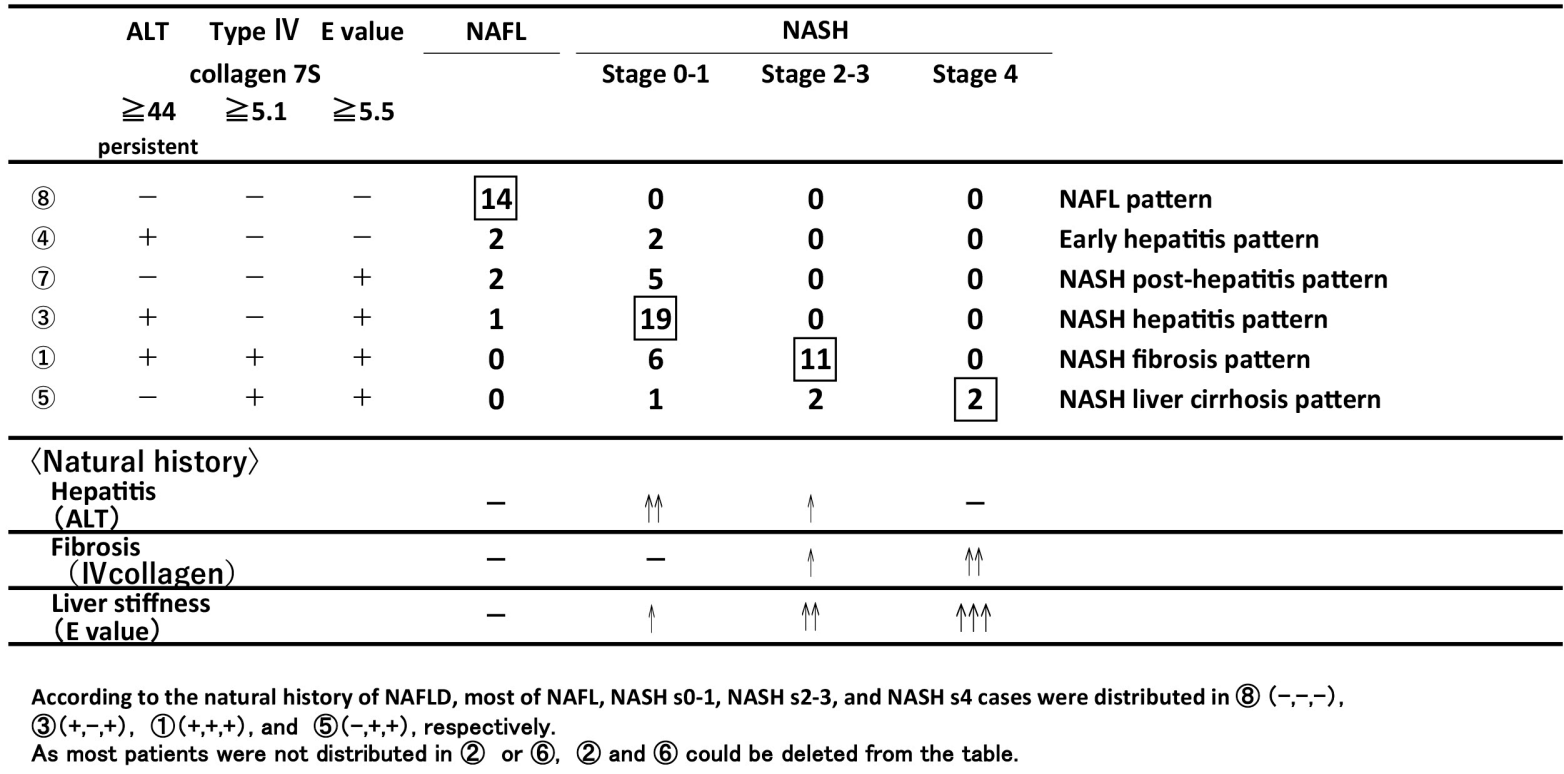
The Case Distributions in the Positive Pattern Recognition System to See the Relationship between Combination Patterns and Histological Classification.

Table 3 summarizes the relationship between the positive patterns using three markers and a pathological condition (NAFL, NASH s0-1, NASH s2-3, and NASH s4). Considering the natural history of NAFL and NASH, the major conditions are as follows: NAFL pattern ⑧ all negative, NASH hepatitis pattern ③ (ALT(+), IV collagen(−), E(+)), NASH fibrosis pattern ① (ALT(+), IV collagen(+), E (+) all positive), and NASH LC pattern ⑤ (ALT(−), IV collagen(+), E (+)). Pattern ④ (ALT(+), IV collagen(-), E(-)) suggested early hepatitis pattern, which showed the stage when NAFL and NASH could not be distinguished. Pattern ⑦ (ALT(−), IV collagen(−), E(+)) was demonstrated by some NASH s0-s1 patients. It is supposed that the NASH patients previously had hepatitis, but it disappeared during the liver function tests. The patients who were pathologically diagnosed with NASH s0-1 demonstrated only positive E value. This condition suggests that E value measurement is sensitive for liver stiffness remained in NASH patients even if the inflammation happened in the past. As previously described, pattern ⑦ (NASH post-hepatitis pattern) would be situated between ④ and ③.

### Comparison of the ACE score with those of previously reported scoring systems

The FIB4 index ^(12), (13)^, NAFLD fibrosis score (NFS) ^(14), (15)^, and BARD score ^(16), (17)^ were previously established and used to predict advanced fibrosis (stages 3 and 4) versus others. On the other hand, the NAFIC score ^(18)^ was used to differentiate between NAFL and NASH patients. The formulae of these scoring systems are presented in Table 4.

**Table 4.**
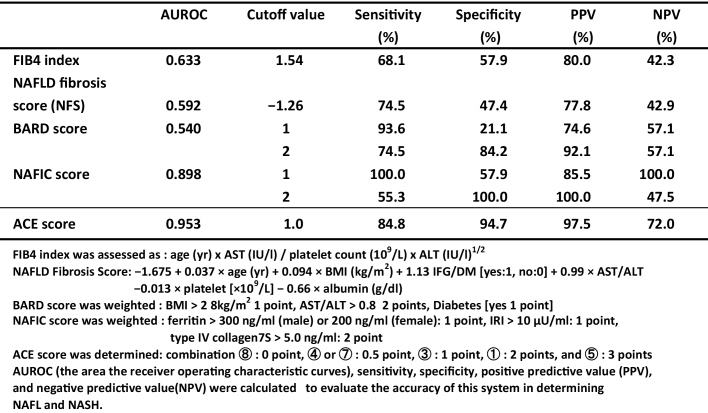
Comparison between the Reported Scoring Systems (FIB4 Index, NFS, BARD Score, and NAFIC Score) and ACE Score for NASH Diagnosis.

To compare the difference between our system and those of others for the differentiation between NAFL and NASH, each positive pattern combination was weighted 0-3 points as ACE score.

ACE score was determined; combination⑧ (NAFL main) was given 0 point, ④ and ⑦ 0.5 point, ③ (early NASH) 1 point, ① (middle NASH) 2 points, and ⑤ (late NASH) 3 points, respectively.

The AUROCs of these scoring systems are plotted (Figure not shown), calculated, and summarized in Table 4. The AUROC was the greatest for the ACE score (0.953), followed by the NAFIC score (0.898), FIB4 index (0.633), NFS (0.592), and BARD score (0.542) (Table 4). The results indicated that the NAFIC score was useful for differentiating NASH from NAFL, but the ACE score was superior to others for distinguishing NAFLD patients.

## Discussion

We could establish the positive pattern recognition system using three markers for differentiating NAFLD. Furthermore, the results of the positive patterns correlated with the histological staging of NASH and NAFL.

Many scoring systems ^(19), (20), (21), (22)^ for the diagnosis of NAFLD instead of liver biopsies have been reported. Because most of them were based on formulas using several biomarkers, the results were expressed as numeric values. However, the concept of our study is different, because the results were expressed as positive pattern combinations based on the existence of chronic hepatitis, progression of liver fibrosis, and degree of liver stiffness. Though the histological evaluation was on steatosis, hepatocyte ballooning, and inflammation as a grading and fibrosis as a staging ^(7)^, major tissue characteristic of image must be basically influenced by inflammation and fibrosis. Consequently, if both chronic hepatitis and progressive fibrosis were properly estimated, it will be possible to decide the stage of NASH along with its natural history.

The main purpose of recent studies was to detect advanced fibrosis in NASH because fibrosis progression is a major risk factor of NASH to determine its prognosis ^(23), (24)^. However, a satisfactory treatment for advanced fibrosis in NASH and NASH・LC has not yet been established. Therefore, it is crucial to make an accurate diagnosis of early NASH and to provide therapeutic interventions (medication, body weight control, and control of insulin resistance) early in order to prevent serious complications.

On the other hand, NASH is not a single disease because it changes from fatty liver to chronic hepatitis, LC, and HCC. According to the clinical observations on NASH patients, hepatitis has been persistent in early stage without treatment, whereas in NAFL patients, hepatitis was not persistent. Furthermore, the ALT values were abnormally high in NASH/hepatitis~ early fibrosis stage, and then they decreased within the normal limit when there was no hepatocyte to break in the decompensated LC stage, since ALT is a deviation enzyme. These are the reasons why ALT is not suitable for use as a marker. As ALT would change along with natural history and be useful for positive pattern recognition, we selected persistent ALT abnormality as a marker.

From the result of the ROC curve (Figure 2), cutoff value (1) was set to 44 IU/L of ALT for distinguishing between NAFL and NASH s0-1/NASH s2-3, whereas cutoff value (2) was set to 36 IU/L for distinguishing between NASH s0-1/NASH s2-3 and NASH s4/LC. The *t*-test revealed significant differences (*P* < 0.003 for (1) and *P* < 0.041 for (2), respectively), presented in Table 2. The positive pattern recognition system, which used ALT, was better in distinguishing not only between NAFL and NASH s0-1/s2-3 but also between NASH s0-1/s2-3 and NASH s4/LC.

There are many fibrosis markers, for example, type IV collagen 7S, hyaluronic acid, mac-2 binding protein, procollagen Type III peptide, and platelet count. Among these markers, type IV collagen 7S is known to show a stable value for liver fibrosis ^(25)^.

As described above (Figure 3-a), the type IV collagen values of NASH s2-3 and NASH s4 were higher than those of NAFL and NASH s0-1. Because AUROC (2) was 0.896 at a cutoff value of 5.1 ng/mL and the *t*-test revealed a significant difference (*P* value < 0.0001, Table 2), type IV collagen 7S was a useful marker for distinguishing between NAFL/NASH s0-1 and NASH s2-3/s4 but not between NAFL and all NASH.

We identified E value as the third predictor for NASH diagnosis, which was obtained by medical imaging not a chemical biomarker. The distribution of the E values significantly changed from NAFL to NASH s0-1, NASH s2-3, and NASH s4 (Figure 3-b). These results indicated that the liver stiffness (E value) would be influenced not only by liver fibrosis but also by chronic inflammation ^(26), (27)^.

The AUROC was 0.926 at a cutoff value of 5.5 KPa, and the *t*-test revealed a significant difference between NAFL and all NASH.

Next, we analyzed the relationship between the positive pattern results using three markers and the histological classification along the natural history of NAFLD.

The total number of combinations was eight. When all NAFL and NASH patients were distributed, patterns ② and ⑥ could be deleted, since it meant that the E value was more sensitive than type IV collagen 7S, as described above.

As presented in Table 3, all NAFL, NASH s0-1, NASH s2-3, and NASH s4 patients had peaks at combinations ⑧, ③, ①, and ⑤, respectively. It should be noted that these NAFLD cases lined up in order and properly distributed in the combination patterns. Moreover, the frequencies of NAFL and NASH cases were well distributed.

The distribution of these histological stages was in line with natural history. Considering the minor condition, pattern ④ (ALT(+), type IV collagen(−), and E(−)) showed that the distinction between NAFL and NASH could not be made, since there was only a pathologically minimal change under early hepatitis.

NASH post-hepatitis pattern ⑦ (ALT(−), type IV collagen(−), and E (+)) showed that the NASH patients previously had hepatitis, but it disappeared when the liver function test was conducted. The reason was that elastography for the liver stiffness measurement made it possible to detect previous traces of hepatitis or minimal fibrosis. Therefore, the E value is useful for detecting seronegative conditions of previous hepatitis or minimal fibrosis.

Our study has several limitations. One is that the number of NAFLD patients was limited, and the percentages of NAFL and NASH s4 patients were 29% and 3%, respectively.

Because the number of NASH s4/LC patients was small, four definite NASH/LC patients without biopsy were added to analyze only statistical results as reference data. We are ready to organize clinical trials to be conducted by cooperative groups and liver centers as well as to include a large number of patients in many locations. Though limitations exist, we would like to propose the idea of a simple system. The positive pattern recognition system based on natural history could predict NASH in NAFLD patients; moreover, the combination patterns exhibited a strong correlation with histological NASH staging.

In conclusion, the use of the diagnostic system for NASH instead of liver biopsy is expected. Considering natural history, three markers were selected: ALT for hepatitis, type IV collagen 7S for liver fibrosis, and E value for liver stiffness.

The point is that the ALT level is almost normal not only in NAFL patients but also in NASH s4 patients. Therefore, the change in ALT is useful for the positive pattern recognition system.

Another important point of this study is that positive pattern recognition taking advantage of the three markers’ strength was adopted because NASH consists of many conditions that exhibit the change of inflammation, fibrosis, and stiffness.

Moreover, the three markers could change independently, and the patterns were different in each stage. Different positive patterns were orderly composed along natural history of NAFL and NASH staging; furthermore, the results demonstrated a strong correlation between positive patterns and histological classification (NAFL, NASH s0-1, NASH s2-3, and NASH s4).

## Article Information

### Conflicts of Interest

None

### Acknowledgement

This study was carried out based on the research collaboration contact between Tenshi Hospital, Kurihara Naika Clinic, and Sapporo Medical University. The authors thank the following individuals for assistance in preparation of this manuscript: Aiko Hayashi and Hiroshi Makino.

### Author Contributions

MT: study concept, design and analysis, drafting of the manuscript, SS: critical revision of the manuscript, statistical analysis, NA: study concept and design, TT, HI: acquisition of data, TI, MY, YK,: critical revision of the manuscript, HN: study supervision. All authors read and approved of the final manuscript.

### Approval by Institutional Review Board (IRB)

Approval code: 147-2021

Name of institution: Tenshi Hospital

Date of approval: November 19, 2021
